# Hand hygiene practice in motherland of semmelweis

**DOI:** 10.1186/2047-2994-4-S1-P308

**Published:** 2015-06-16

**Authors:** P Cserháti

**Affiliations:** 1 Department of Disinfection, National Center of Epidemiology, Hungary

## Introduction

The hand disinfection is the most important part of prevention in healthcare associated infections. This, and one of the WHO guidances (Global Patient Safety Challange) created our test in a Hungarian hospital where we measure the hand hygiene compliance, before and after training. We used three different methods for measurements: microbiological sampling from healthcare workers hands, inspection of the hand hygiene technique, reduction measure of the disinfectant amount.

## Objectives

The purpose of the research was to check the hand disinfection habits of the healthcare workers daily routine. We would like to repeat the whole process in other Hungarian hospitals in 2015. We would like to improve hand hygiene compliance.

## Methods

In case of microbiological sampling, we took samples from healthcare workers dominant hands with sterile bag technique during the workers’ daily routine. 192 employees and 10 hospital department were involved in this survey and we did it before and after training. To identify the microorganisms we used differential agar plates, investigate the cultures with staining and evaluated the colony-forming units with microscope. In parallel inspected of the hand hygiene technique via Hand-in-Scan technology and measured the reduction of the disinfectant amount with help from hospital’s workers. After the employee training we repeated the sampling process.

## Results

After the training we experienced ~30% decrease of total plate counts, the number of Escherichia coli and Pseudomonas aeruginosa decreased by 36% and 26%. Doctors-, Nurses participant rate was 24% and 40% at first time, in case of nurses it increased to 46% at second time.

## Conclusion

After the training we experienced ~30% decrease of total plate counts, the number of Escherichia coli and Pseudomonas aeruginosa decreased by 36% and 26%. Doctors-, Nurses participant rate was 24% and 40% at first time, in case of nurses it increased to 46% at second time.

## Disclosure of interest

None declared.

